# Growth Kinetics and Protective Efficacy of Attenuated ASFV Strain Congo with Deletion of the EP402 Gene

**DOI:** 10.3390/v13071259

**Published:** 2021-06-28

**Authors:** Galina Koltsova, Andrey Koltsov, Sergey Krutko, Natalia Kholod, Edan R. Tulman, Denis Kolbasov

**Affiliations:** 1 Federal Research Centre for Virology and Microbiology, Academician Bakoulov Street 1, 601125 Volginsky, Russia; kolcov.andrew@gmail.com (A.K.); sergejjkrutko@gmail.com (S.K.); natkholod@yandex.ru (N.K.); kolbasovdenis@gmail.com (D.K.); 2 Center of Excellence for Vaccine Research, Department of Pathobiology and Veterinary Science, University of Connecticut, Storrs, CT 06269, USA; edan.tulman@uconn.edu

**Keywords:** ASF, ASFV, recombinant virus, CD2v, EP402R, protective immunity

## Abstract

African swine fever (ASF) is an emerging disease threat to the swine industry worldwide. There is no vaccine against ASF, and progress is hindered by a lack of knowledge concerning the extent of ASFV strain diversity and the viral antigens conferring type-specific protective immunity in pigs. We have previously demonstrated that homologous ASFV serotype-specific proteins CD2v (EP402R) and/or C-type lectin are required for protection against challenge with the virulent ASFV strain Congo (Genotype I, Serogroup 2), and we have identified T-cell epitopes on CD2v which may be associated with serotype-specific protection. Here, using a cell-culture adapted derivative of the ASFV strain Congo (Congo-a) with specific deletion of the EP402R gene (ΔCongoCD2v) in swine vaccination/challenge experiments, we demonstrated that deletion of the EP402R gene results in the failure of ΔCongoCD2v to induce protection against challenge with the virulent strain Congo (Congo-v). While ΔCongoCD2v growth kinetics in COS-1 cells and primary swine macrophage culture were almost identical to parental Congo-a, replication of ΔCongoCD2v in vivo was significantly reduced compared with parental Congo-a. Our data support the idea that the CD2v protein is important for the ability of homologous live-attenuated vaccines to induce protective immunity against the ASFV strain Congo challenge in vivo.

## 1. Introduction

African swine fever (ASF) is an acute viral haemorrhagic disease of domestic swine with a mortality rate close to 100% [1,2,3]. After being imported from Eastern Africa to Georgia [4], ASFV of genotype II has been circulating in Eastern Europe since 2007, in the European Union since 2014, and in Asia since 2018 (OIE). The spread of the disease outside Africa has become a global threat with enormous economic losses for swine-raising countries as well as ecological consequences [5,6].

ASFV is a large icosahedral cytoplasmic virus and the only member of the *Asfarviridae* family [7]. Based on the sequence of the B646L gene encoding the p72 capsid protein, 24 ASFV genotypes were identified [8].

There is no vaccine against ASFV, although protection against homologous virus infection has been observed [9,10,11,12,13,14,15]. Vaccine development and progress in disease control are hindered by a lack of knowledge about the ASFV antigens responsible for the induction of protective immunity and the diversity of these protective antigens in nature.

CD2v is the hemagglutinin of ASFV and has been shown to be involved in protective immunity [16,17,18,19]. A possible role of this protein in the pathogenesis of ASFV infection, in tissue tropism, immune evasion, and enhancement of viral replication in the host has been demonstrated [20,21]. Since its original description, the CD2-like protein encoded by the EP402R gene has been examined during ASF vaccine development. It was shown that immunization of pigs using various viral vectors carrying the CD2v protein can induce strong humoral and cellular immunity [22,23,24], and DNA vaccines expressing the CD2v gene can effectively activate the cytotoxic T lymphocyte (CTL) immune response [25,26,27]. While this immunization did not result in full protection against ASFV, partial protective effects after the virus challenge have been reported in some studies [28,29,30].

We have previously demonstrated that ASFV CD2v (EP402R) and/or C-type lectin (EP153R) proteins are sufficient to mediate serologic specificity determined by a hemadsorption inhibition assay (HAI) and are important for protection against homologous ASFV infection [31,32]. In addition, four discrete regions of the T-cell epitope were identified on CD2v that may indicate a possible involvement of the protein in the formation of the T-cell immune response [33]. Although mutants with a CD2v deletion have been generated for several ASFV strains (BA71, Malawi, and Georgia), the data on the role of the CD2v protein in virus virulence for domestic pigs and in protecting animals from ASF are contradictory [34,35,36,37]. This may be associated with the high genetic variability of the ASFV, so studies of the role of CD2v strains of other genotypes and serotypes could add more insight into the function of this protein.

In this study, we report for the first time the generation of a recombinant ASFV with a deletion of the EP402R gene based on the attenuated ASFV of Genotype I and Serogroup 2. We found that the deletion of the EP402R gene did not significantly affect the ability of the virus to replicate in vitro; however, it resulted in an inability to induce a protective immune response to infection with the parental (i.e., homologous) virulent ASFV strain.

## 2. Materials and Methods

### 2.1. Cell Cultures and Viruses

The attenuated ASFV Congo-a (strain KK262, Genotype I, Serogroup 2) and parental virulent ASFV Congo-v (strain K49, Genotype I, Serogroup 2) were received from the reference collection of the Federal Research Centre for Virology and Microbiology, Russia.

COS-1 cells were kindly provided by C. Gallardo (CISA-INIA, Valdeolmos, Spain). Cells were grown in Dulbecco’s Modified Eagle’s Medium (DMEM) (Gibco, Waltham, MA, USA), supplemented with antimycotic-antibiotic (Gibco) and 10% fetal bovine serum (Gibco) at 37 °C with 5% CO_2_. Primary swine macrophage cultures were prepared from defibrinated blood using Lymphocyte separation media. The cells were cultured in 96-well plates (Corning, New York, NY, USA) (1.3e6/well) containing RPMI 1640 medium supplemented with 30% (*v*/*v*) plasma, 10% (*v*/*v*) fetal bovine serum (Gibco), and antimycotic-antibiotic (Gibco) at 37 °C with 5% CO_2_ for 24 h. Adherent cells were rinsed with the same macrophage medium and used in assays after 48 h.

Virus titration was performed in 96-well plates by visualizing of CPE or hemadsorption in primary swine macrophage cultures. Titers were expressed as mean tissue culture infectious dose (TCID50) or hemadsorption doses (HAD50) respectively, according to the Reed–Muench method [38].

Growth curves of ΔCongoCD2v and parental Congo-a viruses were measured in primary swine macrophage cultures and COS-1 cells using 48-well plates. Cultures were infected with the virus at MOI of 1 and 0.1 (based on virus titer in primary swine macrophage cell cultures). After 1 h of adsorption, the virus was removed, the cells were rinsed with PBS and incubated at 37 °C with 5% CO_2_ in macrophage medium for primary swine macrophage cultures or DMEM with 10% fetal bovine serum for COS-1 cells. The cells were harvested at 0, 24, 48, 72, and 96 h post-infection (hpi) and frozen at ≤−50 °C until lysates were used in qPCR assays [39] and for determination of TCID50/mL titers in primary swine macrophage cell cultures.

### 2.2. Construction of the Recombinant Virus

The attenuated virus Congo-a (KK-262, SG2) was used to generate a recombinant ASFV (ΔCongoCD2v) in which the native CD2v gene (EP402R) was deleted. ΔCongoCD2v was constructed essentially as described previously, using a recombination vector with two recombination arms flanking the GFP reporter gene under control of the ASFV p72 promoter [31]. COS-1 cells were infected with the parental virus (MOI 5) and transfected with recombination vectors in 6-well plates.

The recombinant virus was isolated by limiting dilution and plaque assay in COS-1 cells using fluorescence microscopy. Viral purity was confirmed by Real Time PCR with primers specific for the EP402R gene: 5′-gaagaaatagaaagtccaccacc-3′, 5′-ctgtaaggcttaggaagtaatgg-3′, and Taqman probe 5′-[FAM]gacaccacttccatacatgaacc[RTQ1]-3′. An analysis of viral genomic DNA was also performed by PCR using primers specific for the recombination site (ga2453f:5′-ggaatgtggcatggagatc-3′ and ga4698r: 5′-aagtctttgtaggtttttcgttca-3′), as well as primers specific to the complete EP402R gene. The accuracy of the recombinant sequence was confirmed by DNA sequencing of a fragment of the ASFV ΔCongoCD2v genome of approximately 2200 bp (position in the ASFV Congo-a genome: 63560–65785). Sequencing was performed by the Sanger method on 3130xl Genetic Analyzer (Thermo Fisher, Waltham, MA, USA); the obtained data were analyzed in Sequence Analyzer software (Applied Biosystems, Waltham, MA, USA).

Hemadsorption (HA) assays were used to verify a lack of functional CD2v expression from ΔCongoCD2v-infected cells in vitro by standard methods [31]. Briefly, swine primary macrophages were infected with the parental Congo virus at MOI of 0.1 in the presence of swine red blood cells and assessed for HA by light microscopy at 2 days post-inoculation.

### 2.3. Animal Experiments

Piglets were obtained from a commercial pig farm in Russia and were not vaccinated against any infectious disease. A total of 17 large white pigs (male/female), aged 2–2.5 months (weighing 15–18 kg) and diagnosed as free of specific pathogens (data not shown), were randomly divided into three groups. Each group was housed in an isolated room of the FRCVM animal facility during the entire experiment (including an acclimatization period of one week prior to infection). Pigs from group 1 (*n* = 5) were infected intramuscularly with 10^6^ TCID50 of Congo-a. Pigs from group 2 (*n* = 7) were infected intramuscularly with 10^6^ TCID50 of ΔCongoCD2v. At 21 days dpi, all animals were boosted with the same dose of the same virus. Pigs from group 3 (*n* = 5) were not vaccinated and left to serve as unimmunized controls in the challenge experiments.

Three weeks later, all animals were challenged intramuscularly with 10^3^ HAD50 of virulent Congo-v (strain K-49) and monitored for 30 days. Clinical signs and rectal body temperature were recorded daily throughout the experiment. The clinical assessment of ASF was carried out in 9 different categories (temperature, anorexia, recumbency, skin lesions, swelling of joints, breathing, ocular discharge, digestive findings, neurological disorders), with each scored between 0 and 10 points (Table S1). The sum of points was recorded as a clinical score. Survival and time-to-death were recorded as previously described [32]. To assess viremia and immune responses, blood samples (blood with EDTA and serum) were taken from the jugular vein at 0, 7, 14, 21, 28, 35, and 42 days post-immunization (dpi) and at 3, 5, 7, 14, 21, and 28 days post-challenge (dpc).

During necropsies, five tissues (spleen, liver, lung, mesenteric lymph node, sub-mandibular lymph node) were collected from infected pigs and frozen at −70 °C. Tissue samples were taken for ASFV genome detection.

Quantitative PCR of ASFV in blood and tissues samples was performed as previously described [39]. DNA from blood and tissues samples as well as from infected cell culture was isolated using the ExtractDNA Blood (Evrogen, Moscow, Russia) according to the manufacturer’s instructions.

ASFV ELISA assays were performed as recommended by the manufacturer (IDScreen^®^ African Swine Indirect, Grabels, France) using serum collected at seven-day intervals after the vaccination and challenge. Results are expressed as S/P%.

## 3. Results and Discussion

### 3.1. Construction of the Recombinant Virus

To assess the function of CD2v in ASFV replication in cell cultures and domestic pigs, as well as in the formation of a protective immune response against ASF, a recombinant virus with a deletion of the EP402R gene was constructed. As we have previously demonstrated, the ASF model based on the Congo virus strain is a relevant system to study protective immunity [40]. Overall, 100% of pigs vaccinated with cell-culture attenuated strain Congo-a (KK262) were fully protected from the virulent Congo-v (strain K-49) challenge and showed no clinical symptoms of ASF, while unvaccinated animals died of acute ASF. Here, the attenuated Congo-a virus was used to generate a EP402R gene deletion mutant. Recombinant ΔCongoCD2v was constructed by homologous recombination using a recombination vector with two recombination arms and a GFP reporter gene with a p72 ASFV promoter. The resulting recombinant virus ΔCongoCD2v contained a 1110 bp deletion (nucleotide positions: 64369–65478) and a 917 bp insert compared to the parental strain Congo-a.

**Figure 1:**
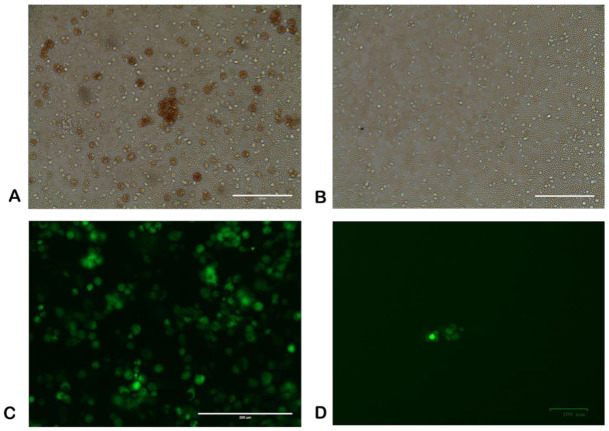
Characterization of ΔCongoCD2v in cell culture. Hemabsorption assays in primary swine macrophages infected with (**A**) parental Congo-v virus, or (**B**) ΔCongoCD2v. (**C**) Fluorescent microscopy of COS-1 cells infected with ASFV ΔCongoCD2v (MOI = 1) at 4 days post-inoculation. (**D**) Fluorescent microscopy of COS-1 cells infected with ASFV ΔCongoCD2v (10^−5^ dilution) at 3 days post-inoculation (plaque assay).

The recombinant virus was purified after eight rounds of limiting dilutions and one round of plaque assay in COS-1 cells (Figure 1D). The virus stock was produced also using COS-1 cells.

The purity of the virus was confirmed by negative real-time PCR using primers specific for the EP402R gene. Moreover, a PCR assay was carried out to amply the complete EP402R gene with primers specific to the gene (Figure S1 lane 4–6). A gel electrophoresis showed that the PCR fragment obtained from the attenuated Congo-a virus matched the size of the complete EP402R gene (Figure S1 lane 5), whereas a PCR fragment was not obtained using recombinant ΔCongoCD2v as a template (Figure S1 lane 4). A PCR analysis using primers flanking the recombination site identified the expected 2.36 kb fragment in parental Congo-a virus (Figure S1 lane 2). While a 2.15 kb fragment was amplified from genomic DNA of the recombinant ΔCongoCD2v virus, as expected, due the EP402R gene being replaced by the EGFP gene under control of the p72 ASFV promoter (Figure S1 lane 1). Recombinant ΔCongoCD2v did not exhibit hemadsorption in macrophage cell cultures, which also indicated the viral purity and efficient deletion of the EP402R gene (Figure 1A,B).

Sequencing was used to confirm that the deletion occurred at the right genome location and there were no mutations at the recombination site. The sequencing results confirmed the absence of the EP402R gene and any additional mutations in the ΔCongoCD2v genome fragment.

### 3.2. Replication of Recombinant Virus in Cell Cultures

Deletion mutant virus ΔCongoCD2v showed the ability to grow efficiently in primary swine cell cultures (swine macrophage culture and born marrow cells) as well as in COS-1 cells (Figure 1C). As expected, unlike the Congo-a virus, replication of the recombinant virus in primary cultures (in the presence of erythrocytes) was not accompanied by hemadsorption.

Evaluation of the effect of CD2v protein on viral replication in vitro was carried out in primary swine macrophage cultures and COS-1 cells using multistep growth curves. The cultures were infected with the parental or recombinant virus at an MOI of 1 or 0.1. At 0, 24, 48, 72, 96, and 120 hpi, virus yield was quantified by qPCR. A multiplicity of infection of 0.1 was used to allow multiple replication cycles during the experiment.

**Figure 2:**
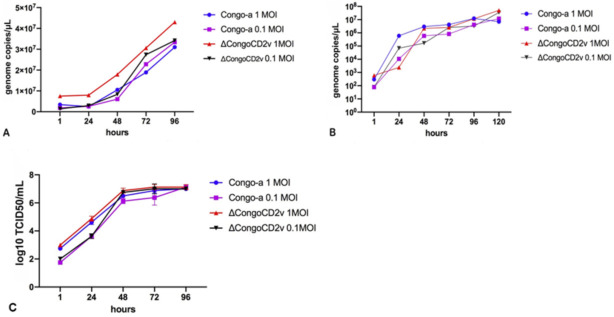
Kinetics of in vitro growth of parental Congo-a and recombinant ASF viruses in primary swine macrophage cultures and COS-1 cells. (**A**): multistep growth curves in primary swine macrophage culture. (**B**): multistep growth curves in COS-1 line cells. (**C**): multistep growth curves in primary swine macrophage culture, expressed in log10 TCID50/mL. An analysis was conducted using Graphpad Prism software version 8.0.1.

Our results demonstrated that growth kinetics of ΔCongoCD2v were almost identical to those of the parental Congo-a virus in primary swine macrophage culture and in COS-1 cells (Figure 2A,B). To confirm the results obtained, two independent experiments were carried out to assess the in vitro kinetics of virus growth in primary swine macrophage cell cultures infected with both viruses at an MOI of 1 or 0.1. Infected cells were used to determine the titers by TCID50/mL in primary swine macrophage cell cultures. No significant differences in virus titers were observed until the end of the experimental period (96 hpi) (Figure 2C).

These results showed that deletion of the EP402R gene did not significantly affect the ability of the virus to replicate in primary and continuous cell cultures. Our data on the CD2v protein of the ASFV strain Congo-a are fully consistent with the data obtained for the ASFV strain BA71 [34] and ASFV field isolates in Malawi [35] and Georgia 2010 [36,37], and confirm that this protein does not significantly affect virus replication in primary swine cell cultures.

### 3.3. Animal Experiments

A classic vaccination/challenge experiment was designed to test the kinetics of infection of parental and recombinant viruses in vivo and to assess the ability of ASFV ΔCongoCD2v to induce a protective immune response.

**Figure 3:**
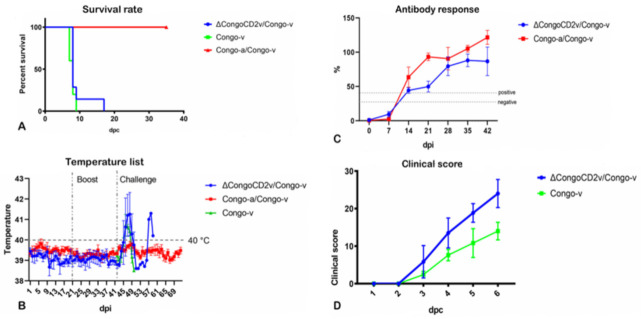
Kinetics of lethality (**A**) and body temperature (**B**) in pigs inoculated with ASFV strain Congo-a, strain ΔCongoCD2v, and in mock-vaccinated pigs. The medium-dashed line represents the fever-cutoff (40 °C) (**B**). Anti-ASFV antibody responses (**C**) measured using ELISA in pigs immunized with ASFV strain ΔCongoCD2v and strain Congo-a at 0–42 dpi (cut-off 40%). Clinical scores (**D**) in pigs immunized with ASFV strain ΔCongoCD2v and in mock-vaccinated pigs following challenge with virulent ASFV Congo-v at 0–6 dpc. Pigs were immunized with Congo-a (strain KK-262) (red line) or ΔCongoCD2v recombinant virus (blue line), mock-vaccinated pigs (green line). Animals were challenged with virulent Congo-v (strain K-49). The data of body temperature, anti-ASFV antibody response, and clinical score are shown as average values and their SD in each of the groups. Analysis was conducted using Graphpad Prism software version 8.0.1.

Transient fever was detected in some pigs immunized with Congo-a at 5–7 days dpi (Figure 3B). The ASFV genome was detected by qPCR in animals immunized with Congo-a at 3 to 42 dpi. Similar to its Congo-a parental strain, the ΔCongoCD2v deletion mutant was attenuated upon intramuscular infection of pigs at doses of 10^6^ TCID50. Animals immunized with ΔCongoCD2v remained clinically normal, including no increase in body temperature (>40 °C) (Figure 3B). In contrast to pigs immunized with Congo-a, in pigs immunized with ΔCongoCD2v the ASFV genome in blood was not detected up to 42 dpi.

The absence of viremia in animals inoculated with the ΔCongoCD2v virus was confirmed by testing blood samples in swine macrophage cell culture on different days post-inoculation. This seems to indicate that although the deletion of the EP402R gene did not significantly affect the ability of the virus to replicate in vitro in swine macrophages, replication in vivo of this deletion virus was significantly reduced. Our data are consistent with those of Gladue et al. (2020) [37] and indicate that the expression of CD2v protein has some relationship with the spread of ASFV in domestic pigs. Interestingly, the deletion of EP402R from the genome of virulent isolates resulted in only a moderate decrease in virus titers [35,36,37]. It is unclear whether these observed differences are related to genetic differences in ASFV strains or other unidentified factors of host–virus interaction.

The presence of anti-ASFV antibodies in the serum of animals immunized with Congo-a and ΔCongoCD2v and prior to the challenge was tested by ELISA assay. In general, the immunized animals were found to be positive (S/P > 40%) for anti-ASFV antibodies 14–42 days after inoculation with attenuated viruses (Figure 3C). In only one animal immunized with ΔCongoCD2v, the ELISA test showed an S/P of less than 40%, but was still higher than 30% (39.8%) on day 14. The optical density (OD) values gradually increased during the experiment. Mean OD values, and thus calculated S/P% values, of Congo-a immunized animal sera were slightly higher than were mean OD and S/P% values of sera from ΔCongoCD2 immunized animals; however, this difference was not statistically significant. Similar OD values and the dynamics of accumulation of anti-ASF antibodies in the blood serum of animals of both groups may indicate the replication of the recombinant virus in vivo, despite the absence of the virus (virus genome) in the blood of pigs. It has been noted that the induction of ASFV-specific antibodies is directly related to the presence of the replication of attenuated viruses [37,41,42].

All animals from the two immunized groups and the control group (mock-vaccinated pigs) were challenged intramuscularly with 10^3^ HAD50 of virulent Congo-v (strain K-49) on 42 dpi. Back-titration of the material used to challenge the animals showed that the pigs were inoculated with material containing 1 × 10^3^ HAD50/mL, which matched the expected titer.

After infection, the animals from the control group developed typical clinical symptoms of ASF including hyperthermia, depression, and anorexia. Nevertheless, bleeding from the nose or rectum was not observed. The pigs in the control group were dead or found in a moribund state and euthanized at 7–9 dpc.

In animal experiments, it was observed that ASFV Congo-a induces strong protective immunity when challenged with the virulent Congo-v. All recovered pigs from the immunized Congo-a group, regardless of their gender and body weight, showed no clinical signs associated with the disease until the end of the observation period (30 dpc) (Figure 1A). All animals showed good health, appetite, and activity.

In contrast, animals immunized with the recombinant ΔCongoCD2v virus were not protected from challenge with the virulent Congo-v. Mortality and onset of clinical disease, including fever and other clinical signs, were comparable to those seen in the mock-vaccinated controls (Figure 1A,B,D). There were no clear differences in time of death (or humanistic euthanasia) between animals immunized with ΔCongoCD2v or control animals (Table 1). However, one animal from the ΔCongoCD2v/Congo-v group died at 17 dpc. The clinical signs of ASF were more pronounced in animals immunized with the recombinant ΔCongoCD2v virus than in control animals (Figure 3D). While this is an interesting result, a more detailed study is required to elucidate these findings. Viraemic loads with high titers were detected and persisted until death. In addition, the number of ASFV genome copies in the blood and organs of animals immunized with ΔCongoCD2v or the control animals did not show clear differences (Table 1). The ASFV genome was not detected in blood and organ samples of animals immunized with Congo-a.

Importantly, only very low or undetectable amounts of the recombinant ASFV genome were detected in the organs of animals immunized with ΔCongoCD2v. Recombinant ASFV DNA was detected by Real Time PCR with primers specific for the EGFP reporter gene [43] present in ΔCongoCD2v and absent in Congo-v. Ct values varied from 33 to 45 (Table S2). Due to the high titer of the virulent Congo-v virus post-challenge in organs of animals immunized with ΔCongoCD2v, it was not possible to isolate and confirm the presence of a replication-competent, recombinant ΔCongoCD2v post-challenge. However, these results confirmed the replication of the recombinant virus in vivo, despite the absence of the virus in the blood of immunized pigs.

Thus, clear statistical differences were shown between the attenuated Congo-a and recombinant ΔCongoCD2v in the induction of a protective immune response against the homologous virulent Congo challenge. These data support the concept that the CD2v protein is important for the ability of a live-attenuated Congo-a vaccine to induce protection against a homologous ASFV challenge, consistent with data using other ASFV strains [37]. While our previous data using chimeric ASFV indicate that CD2v/C-type lectin are also important serotype-specific ASFV protective antigens [32,33], a lack of protective efficacy here with ΔCongoCD2v may largely instead be due to the lower level of replication of an EP402R-deleted mutant in vivo as observed elsewhere [37]. The critical protective antigens and host immune mechanisms responsible for protecting against ASF remain unclear and need to be elucidated.

In summary, we have demonstrated that the deletion of the EP402R gene from attenuated strain Congo-a did not significantly affect its ability to replicate in primary and continuous cell cultures, but resulted in the inability to induce in vivo a protective immune response against the challenge with the homologous and virulent ASFV strain Congo.

## Figures and Tables

**Table 1:** Pig survival, fever, and maximum viral load following infection with Congo-a and ΔCongoCD2v and challenge with 10^3^ HAD50 ASFV Congo-v.

Group.	No of Animals	Mortality		Fever		Pre-Challenge Serology (%)	Pre-Challenge Max Viral Load	Post-Challenge Max Viral Load (Genome Copies/mL)	Max Viral Load in Organs (Genome Copies/mL)
		%	TTD (SE)	%	TTF (SE)				
**ΔCongoCD2v/Congo-v**	7	100	9.43 (1.26)	100	5.57 (1.6)	100	Neg	1.034 × 10^8^	9.178 × 10^8^
Congo-a/Congo-v	5	0		0		100	7.0 × 10^2^		
Congo-v	5	100	7.8 (0.374)	100	3.6 (0.25)			1.32 × 10^9^	5.838 × 10^8^

TTD, Mean time-to-death in days post-challenge, with SE in parentheses. TTF, Mean time-to-fever in days post-challenge, with SE in parentheses.

## Data Availability

The data presented in this study are available on request from the corresponding author.

## Supplementary Materials

The following are available online at https://www.mdpi.com/article/10.3390/v13071259/s1, Figure S1: Analysis of the EP402R gene deletion in genomic viral DNA by PCR, Table S1: Clinical Scoring of swine inoculated with ASFV, Table S2: Detection of EGFP gene in organs and lymph nodes of ΔCongoCD2v and Congo-a immunized pigs following the challenge with ASFV Congo-v.
