# Supplementary material for: Growth Kinetics and Protective Efficacy of Attenuated ASFV Strain Congo with Deletion of the EP402 Gene
Source: Viruses. 2021 Jun 28;13(7):1259. doi: 10.3390/v13071259 (PMC8309992; doi:10.3390/v13071259)
Supplement: Supplementary file 1 [file viruses-13-01259-s001.zip › viruses-1217481-supplementary.pdf]

viruses

Supplementary materials

Table S1. Clinical Scoring of swine inoculated with ASFV.

Characteristic	Score (points)	
**Anorexia**		
	**1-4**	Reduced eating
	**5-8**	Only picking at food
	**9-10**	Not eating
**Recumbency**		
	**1-3**	Lethargic
	**4-5**	Animal gets up only when touched
	**6-8**	Gets up only slowly when touched
	**9-10**	Remains recumbent when touched
**Neurologic signs**		
	**0**	Normal
	**1-10**	Neurological disorders (ataxia, paralysis, convulsion) depending on the occurrence and severity of signs
**Skin lesion**		
	**0**	Normal
	**1-10**	Skin erythema, edema, and hemorrhages depending on combined assessment
**Swelling of joints**		
	**0**	Normal
	**1-5**	Joint swelling depending on the severity of signs
	**5-10**	Severe swelling with lameness depending on the severity of signs
**Breathing**		
	**1-5**	Labored and/or coughing depending on the severity of signs
	**5-10**	Severe depending on the severity of signs
**Ocular discharge**		
	**1-4**	Mild
	**5-10**	Moderate
**Digestive findings**		
	**0**	Normal
	**1-3**	Mild diarrhea for less than 24 h
	**4-6**	Moderate findings, e.g., diarrhea and vomiting for more than 24 h
	**7-10**	Severe, bloody diarrhea and/or bloody urine
**Body Temperature**		
	**0**	38-40.2°C
	**3-5**	40.3-40.5°C
	**6-8**	40.6-41°C
	**9-10**	> 41.0°C

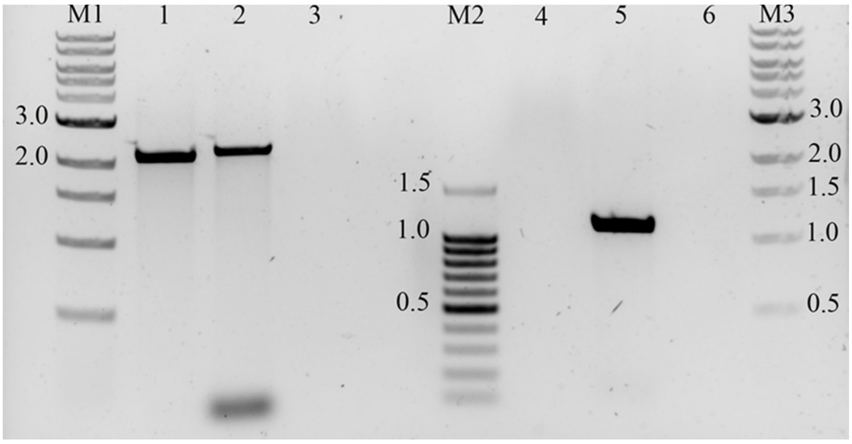

Figure S1. Analysis of the EP402R gene deletion in genomic viral DNA by PCR. Viral DNA was extracted from parental Congo-a virus and the recombinant ΔCongoCD2v virus. Specific PCR fragments were amplified and analyzed by electrophoresis in 1.5% agarose gel. Lanes 1–3: PCR with primers flanking the recombination site; lanes 4–6: PCR of the complete EP402R gene. The following viral genomic DNAs were used as templates: recombinant ΔCongoCD2v virus (lanes 1 and 4), parental Congo-a virus (lanes 2 and 5), no template (lanes 3 and 6). Lanes M1 and M3 contain a 1 kb DNA ladder, and lane M2 contains a 100 bp DNA ladder.

Table S2. Detection of EGFP gene in organs and lymph nodes of ΔCongoCD2v and Congo-a immunized pigs following the challenge with ASFV Congo-v.

Group	# Animal	CT Value of Real-time PCR				
		Spleen	Liver	Lung	Mesenteric Lymph Node	Sub-mandibular Lymph Node
ΔCongoCD2v	1/1	40,32	42,21	33,01	43,17	41,27
	1/2	Neg	Neg	Neg	42,4	43,54
	1/3	Neg	Neg	Neg	44,89	Neg
	1/4	Neg	Neg	44,54	Neg	Neg
	1/5	41,32	Neg	39,87	42,67	42,54
	1/6	43,1	Neg	43,78	41,32	42,76
	1/7	43,98	Neg	Neg	Neg	44,91
Congo-a	2/1	Neg	Neg	Neg	Neg	Neg
	2/2	Neg	Neg	Neg	Neg	Neg
	2/3	Neg	Neg	Neg	Neg	Neg
	2/4	Neg	Neg	Neg	Neg	Neg
	2/5	Neg	Neg	Neg	Neg	Neg

Neg – no CT value.
